# Calorie Restriction Hysteretically Primes Aging *Saccharomyces cerevisiae* toward More Effective Oxidative Metabolism

**DOI:** 10.1371/journal.pone.0056388

**Published:** 2013-02-11

**Authors:** Erich B. Tahara, Fernanda M. Cunha, Thiago O. Basso, Bianca E. Della Bianca, Andreas K. Gombert, Alicia J. Kowaltowski

**Affiliations:** 1 Departamento de Bioquímica, Instituto de Química, Universidade de São Paulo, São Paulo, São Paulo, Brazil; 2 Escola deArtes, Ciências e Humanidades, Universidade de São Paulo, São Paulo, São Paulo, Brazil; 3 Departamento de Engenharia Química, Escola Politécnica, Universidade de São Paulo, São Paulo, São Paulo, Brazil; Baylor College of Medicine, United States of America

## Abstract

Calorie restriction (CR) is an intervention known to extend the lifespan of a wide variety of organisms. In *S. cerevisiae*, chronological lifespan is prolonged by decreasing glucose availability in the culture media, a model for CR. The mechanism has been proposed to involve an increase in the oxidative (*versus* fermentative) metabolism of glucose. Here, we measured wild-type and respiratory incompetent (ρ^0^) *S. cerevisiae* biomass formation, pH, oxygen and glucose consumption, and the evolution of ethanol, glycerol, acetate, pyruvate and succinate levels during the course of 28 days of chronological aging, aiming to identify metabolic changes responsible for the effects of CR. The concomitant and quantitative measurements allowed for calculations of conversion factors between different pairs of substrates and products, maximum specific substrate consumption and product formation rates and maximum specific growth rates. Interestingly, we found that the limitation of glucose availability in CR *S. cerevisiae* cultures hysteretically increases oxygen consumption rates many hours after the complete exhaustion of glucose from the media. Surprisingly, glucose-to-ethanol conversion and cellular growth supported by glucose were not quantitatively altered by CR. Instead, we found that CR primed the cells for earlier, faster and more efficient metabolism of respiratory substrates, especially ethanol. Since lifespan-enhancing effects of CR are absent in respiratory incompetent ρ^0^ cells, we propose that the hysteretic effect of glucose limitation on oxidative metabolism is central toward chronological lifespan extension by CR in this yeast.

## Introduction

Calorie restriction (CR) is an intervention well demonstrated to enhance the lifespan of a wide range of organisms, from yeasts to laboratory rodents (reviewed in refs. [1]–[5]). The alterations promoted by CR are clearly pleiotropic and, as a result, many groups have focused on pinpointing CR effects central toward the extension of lifespan. In this sense, the unicellular eukaryote *S. cerevisiae* has proven to be a valuable research tool, since it is easy to manipulate genetically and metabolically, and presents significantly shorter lifespan than most laboratory model systems. Furthermore, *S. cerevisiae* responds to a decrease in glucose concentration in the culture media (a model for CR) with an increase in both replicative lifespan (a measure of the number of daughter cells generated by a mother cell) and chronological lifespan (a measure of the survival time or viability during the stationary phase, reviewed in refs. [6]–[9]).

Lin et al. [10] first proposed that the effect of CR in yeast replicative lifespan was dependent on an increase in respiratory rates promoted by this intervention, although later results questioned the specific need for respiratory enhancements for the extension of replicative lifespan [11], [12]. Subsequently, many different groups and experimental approaches clearly demonstrated enhanced respiratory rates are necessary for the increment of chronological lifespan promoted by CR. Evidence in this sense includes (i) the observation that respiratory-incompetent cells due to defective mitochondrial DNA (ρ^0^ cells) or defects in nuclear respiratory genes do not respond to CR with an extension in chronological lifespan [13]; (ii) results indicating that enhancing respiratory activity by increasing *HAP4* expression, adding uncouplers or nitric oxide donors to cell cultures enhances chronological lifespan [14]–[16]; (iii) the finding that *Kluyveromyces lactis*, a yeast which does not enhance respiratory rates when glucose is limited, does not respond to CR with an increase in chronological lifespan [17]; and (iv) the finding that the uptake of ethanol, an exclusively respiratory substrate in yeast, plays a central role in chronological lifespan extension by CR in *S. cerevisiae* [18].

In *S. cerevisiae*, the increment in respiratory rates observed by CR is associated with the limitation of glucose in the culture media. In this Crabtree-positive yeast, higher glucose levels repress oxidative metabolism and favor glucose fermentation to ethanol, while low glucose levels allow for the oxidation of glucose to CO_2_. As a result, glucose concentrations determine the metabolic fate of pyruvate [19], [20]. In this sense, enhancement of respiration due to CR could be a particularity of this yeast model of aging. However, a wealth of evidence supports the idea that CR also stimulates respiratory rates in animals, including *C. elegans*, laboratory rodents and humans [21]–[29]. In animals, the increase in respiratory activity promoted by CR is the result of enhanced mitochondrial biogenesis and involves nitric oxide signaling initiated by adiponectin [21], [30]. Altogether, these findings reinforce the central importance of augmented respiratory activity in the lifespan-enhancing effects of CR.

However, many questions still remain regarding the role of respiratory metabolism in lifespan. In *S. cerevisiae*, limiting glucose availability apparently enhances respiratory metabolism in the early growth and stationary phases, but little is known about long-term responses throughout the chronological lifespan of this yeast [31]. Thus, the aim of this work was to quantitatively monitor time-dependent changes in energetic metabolism of *S. cerevisiae* over a wide aging window and uncover how it is modulated by CR and mitochondrial respiration. In doing so, we uncovered a novel, hysteretic, glucose-mediated effect on respiratory activity that affects long-term *S. cerevisiae* survival.

## Materials and Methods

### 1. *S. cerevisiae* cells: Parental strains and ρ^0^ mutants

**Figure 3:**
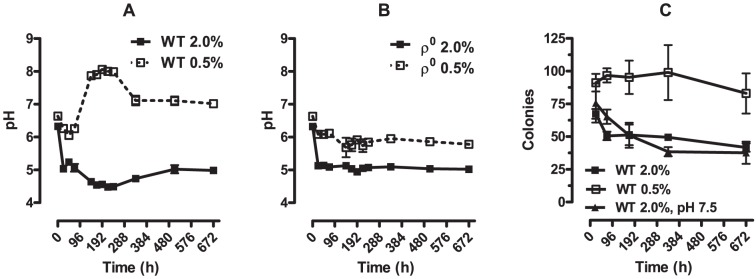
Extracellular pH changes during chronological lifespan. Extracellular pH (Panels A and B) was determined as described in Materials and Methods in the culture media of WT and ρ^0^ (Panels A and B, respectively) *S. cerevisiae* over the course of chronological aging. In Panel C, the chronological lifespan of WT cells cultured under control and CR conditions was followed over time. Hepes (100 mM) was added to maintain the pH at 7.5 where indicated. Values are expressed (means±SEM) indicate the number of colonies formed from 100 cells plated onto solid YPD media.

The parental strain of *S. cerevisiae* used in this study was BY4741 (MATa, *his3Δ1*, *leu2Δ0*; *met15Δ0*, *ura3Δ0*), except in Fig. 3C, in which the RJD1144 strain was used. ρ^0^ mutants were obtained through the identification, isolation and characterization of spontaneous respiratory incompetent colonies, as described elsewhere [13]. Briefly, after growth of WT cells in liquid YPD for 20 h, cells were plated onto solid YPD and this plate was replicated onto YPEG, a respiratory-selective medium. Respiratory incompetent colonies were then identified and isolated. The ρ^0^ phenotype of selected colonies was confirmed by mating them with *S. cerevisiae* mit^-^ strains containing point mutations in mitochondrial genes. After diploid selection based on heterozygous auxotrophy complementations, no reversion of respiratory incompetence was observed. We then selected one isolated colony and further characterized it by following its growth curve (which did not exhibit pos-diauxic biomass formation) and by monitoring the exhaustion of aerobic metabolites from culture media.

### 2. Culture conditions, media and cell storage

Culture medium used for the experimental procedures was liquid YPD (2.0% peptone, 1.0% yeast extract and 2.0% or 0.5% glucose; the latter for CR) and cells were stored in solid YPD (standard liquid YPD supplemented with 2.0% bacteriological agar) at 6°C or in standard liquid YPD supplemented with 20% glycerol, at −80°C. All media were autoclaved at 121°C for 20 min. Cell cultures were performed in aseptic erlenmeyers sealed with cotton/gauze stoppers containing volumes of culture medium ranging from 80 to 100 mL, at 30°C, for 28 days, in an incubator operating under constant 200 rpm orbital agitation. The pre-cultures were carried out for ∼18 hours and the number of inoculated cells per mL of fresh medium to initiate all cultures was 1⋅10^5^ [32].

### 3. Oxygen consumption

Oxygen consumption in intact WT *S. cerevisiae* cells was followed using a Clark electrode operating with continuous stirring at 30°C in 1–200 mL culture aliquots. The absorbance at 600 nm (Abs_600_) was determined for each experimental sample, to calculate yeast biomass (as described in *item 6*, *Materials and Methods*). Data are expressed as nmols of oxygen per g of biomass per minute.

### 4. Separation, analysis and quantification of extracellular *S. cerevisiae* metabolites

Analytes in YPD culture medium were separated using a Bio-Rad Aminex HPX-87H column (operating at 60°C) coupled to an HPLC system with 5 mM H_2_SO_4_ as the mobile phase, at a flow rate of 0.6 mL^.^min^−1^. The analysis of extracellular metabolites was conducted using a Waters 2414 refraction index detector (operating at 50°C) and a Waters UV/Vis 2489 absorbance detector (operating at 214 nm). The quantification of metabolites used Empower Chromatography Data software (Waters). Culture media aliquots (1 ml) were taken from both WT and ρ^0^ *S. cerevisiae* at the initial time (0 h), at 6, 12, 18, 24, 30, 36, 42 and 48 hours, and also at culture days 3, 6, 7, 8, 9, 10, 14, 21 and 28. Once collected, aliquots were immediately filtered with Millex GV filter units with a 0.22 μm Durapore membrane (Millipore) to remove cellular contents and then stored in centrifuge microtubes at −20°C until analysis, carried out within 4 weeks. Analytes studied were glucose [retention time (rt)  =  9.39 min], succinate (rt  =  12.33 min), glycerol (rt  =  13.81 min), acetate (rt  =  15.76 min) and ethanol (rt  =  22.29 min), all detected by refraction index, and pyruvate (rt  =  9.58 min), detected by UV absorbance. The standards containing 10.0 g^.^ L^−1^ glucose, 5.0 g^.^ L^−1^ ethanol and glycerol, and 1.0 g^.^ L^−1^ pyruvate, succinate and acetate, or 5.0 g^.^ L^−1^ glucose, 2.5 g^.^ L^−1^ ethanol and glycerol, and 0.5 g^.^ L^−1^ pyruvate, succinate and acetate used to obtain calibration curves were analyzed at the beginning, middle and end of every batch of chromatographic analysis.

### 5. Cell growth and extracellular pH

Time courses of cell growth and extracellular pH curves for WT and ρ^0^ *S. cerevisiae* were constructed by determining Abs_600_ and the pH from the extracellular environment at the same times in which aliquots for analysis of extracellular metabolites were removed. Whenever necessary, dilutions were made so that the spectrophotometric reading was lower than 0.6.

### 6. Biomass determination

Biomass determination for physiological parameter calculations was performed according to Olsson et al. [33] with minor modifications. A volume ranging from 3 to 20 mL of culture medium containing *S. cerevisiae* was filtered through a Millipore 0.45 μm pore filter membrane. The membrane filters were previously stored in a drying oven at 85°C for 8 h, after which they had their individual masses measured. After filtration, membrane filters were removed from the glass filter unit and stored in the same drying oven at 85°C for a further 8 h. Their masses were measured once again, and the values of yeast dry mass – that is, the biomass – were calculated. After ten repetitions of this procedure at points throughout the 28 day culture period we determined the highly reproducible value of 0.194 mg of dry BY4741 *S. cerevisiae* per mL of culture medium per unit of Abs_600_, regardless of the growth phase.

### 7. Chronological lifespan determination

Chronological lifespan was accessed through colony-forming ability over time. After 16 h and 7, 14, 21 and 28 d of growth, we transferred a 2 mL aliquot of from each culture to a sterile centrifuge conic tube and added 3 mL of sterile ultra-purified distilled water. The suspension was centrifuged for 1 min at 1000 x g, 25°C, and the supernatant was discarded. The washing procedure was repeated. The cells were resuspended in 2 mL of sterile ultra-purified distilled water and the absorbance at 600 nm (Abs600) was determined. Serial dilutions to a final Abs600 of 0.2, 0.02, 0.002 and 0.0002 were conducted and 50 μL of the last dilution (containing 100 cells) were added to YPD plates and incubated for 72 h to promote cellular growth, after which the number of colonies was counted [13], [32]. Results are indicated as the absolute number of colonies counted.

### 8. Calculation of physiological parameters

Collected chromatographic data were used to plot time based graphs. Linear regressions used for the determination of physiological parameters were obtained using OpenOffice.org Calc 3.2.1 (Oracle) software. The minimum linear regression coefficient accepted for data analysis was 0.9.

### 9. Maximum specific growth rate in glucose and ethanol/glycerol

To determine the maximum specific growth rate (μ^max^; h^−1^) supported by glucose (μ_Glu_^max^) and ethanol/glycerol (μ_EtOH+Gly_^max^), we first generated a natural logarithm plot of cell concentration (biomass; ordinate) *versus* time (abscissa). The μ^max^ for each substrate corresponds to the slope of the regression line obtained with the points belonging to the linear segment of the growth curve. This portion corresponds to the exponential phase of cell growth promoted by the use of each of the substrates [34]. Time intervals used for the calculation of μ_Glu_^max^ and μ_EtOH+Gly_^max^ were determined by Abs_600_ and are shown in Table 1, as well as the time interval to the beginning of the metabolism of ethanol and glycerol after the exhaustion of glucose. The maximum specific growth rate on ethanol and glycerol are shown as a single index, since the consumption of these two substrates was parallel.

**Table 1:** Time intervals (Δt) used to calculate μ_Glu_^max^, μ_EtOH+Gly_^max^ and ethanol and glycerol consumption after glucose exhaustion in WT and ρ^0^ *S. cerevisiae*.

	Δt to μ_Glu_^max^	Δt to μ_EtOH+Gly_^max^	Δt between μ_Glu_^max^ and μ_EtOH+Gly_^max^
**WT 2.0%**	0 to 18 h	30 to 48 h	12 h
**WT 0.5%**	0 to 12 h	18 to 42 h	6 h
**ρ^0^ 2.0%**	0 to 24 h	—	—
**ρ^0^ 0.5%**	0 to 18 h	—	—

### 10. Determination of substrate-to-biomass conversion factors

To determine the substrate-to-biomass conversion factors during exponential growth phases, or the cell yield (Y_X/S_^exp^, in g cells/g substrate) for glucose (Y_p/Glu_^exp^, in g cells/g glucose) and ethanol/glycerol (Y_X/EtOH+Gli_^exp^, in g cells/g ethanol + glycerol), the values of Abs_600_ were converted into biomass (as previously described in this section). The slope of the regression line obtained for the cell concentration (g cells/L; ordinate) *versus* substrate concentration (g glucose/L or g ethanol + glycerol/L; abscissa) graph corresponds to the substrate-to-biomass conversion factor. Time intervals of biomass curves used for these calculations correspond to those listed in Table 1.

### 11. Determination of substrate-to-product conversion factors

To determine the substrate-to-product conversion factor, or the product yield (Y_X/S_^exp^, in g product/g substrate), we first generated a graph of the concentration of the product (g product/L; ordinate) *versus* the concentration of the substrate (substrate g/L). The slope of the regression line obtained corresponds to Y_P/S_. Subsequently, glucose-to-ethanol (Y_EtOH/Glu_^exp^) and glucose-to-glycerol (Y_Gli/Glu_^exp^) conversion factors were obtained. Time intervals used for determining these parameters are those in which glucose consumption and the generation of products (Table 1) were observed concomitantly. The conversion of glucose-to-acetate, glucose-to-pyruvate and glucose-to-succinate could not be calculated since theses metabolites were only detected after complete exhaustion of glucose from the culture media.

### 12. Determination of maximum specific consumption of glucose rate and maximum specific rate of product formation

Maximum specific consumption rates of substrates (r_c_^max^; g substrate/g cells^.^h) and maximum specific formation rates of products (r_f_^max^) were calculated using equations 1 and 2, respectively. We thus determined the maximum specific consumption rates of glucose (r_cGlu_^max^; g glucose/g cells^.^h); of ethanol and glycerol (r_cEtOH+Gly_^max^, ethanol + glycerol in g/g cells^.^h), and the maximum specific rate of ethanol (r_fEtOH_^max^; g ethanol/g cells^.^h) and glycerol (r_fGly_^max^; g glycerol/g cells^.^h) formation.



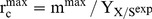
(1)




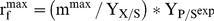
(2)


### 13. Graphs and statistical analysis

Graph generation and statistical analysis were performed using GraphPad Prism 5.00 (GraphPad Software, Inc.). Results are expressed as mean±mean error.

## Results

### 1. Glucose levels in the early hours of culture affect long-term oxygen consumption rates

Chronological aging in *S. cerevisiae* involves growth in batch cultures over the course of several weeks, a period in which large metabolic changes are expected to occur. CR in *S. cerevisiae*, which involves decreasing the initial availability of glucose in the growth media from the typical level of 2.0% (referred to here as control) to 0.5% (CR), has been previously suggested to promote a shift from fermentative to oxidative metabolism in the early culture phase [10], [15], [32]. Furthermore, respiratory integrity has been found to be essential for the chronological lifespan extension of *S. cerevisiae* promoted by CR [13], [14], [35].

**Figure 1:**
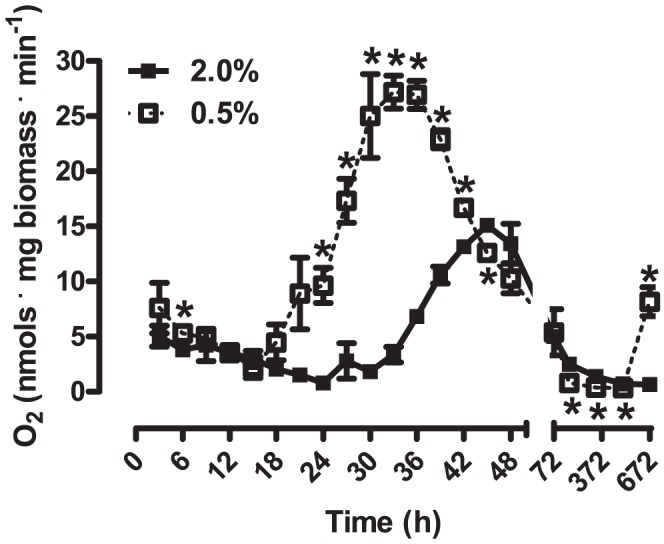
Oxygen consumption rates vary during WT *S. cerevisiae* chronological lifespan. Intact cellular respiratory rates of WT *S. cerevisiae* cultured in 2.0% glucose (▪) or 0.5% glucose (□) were measured as described in *Materials and Methods*. *p<0.05 *vs.* 2.0% WT (unpaired Student's *t* test).

In order to understand how glucose levels in culture and oxidative metabolism are related to chronological aging, we quantified oxygen consumption rates in intact WT *S. cerevisiae* cells cultured under control and CR conditions in YPD during 28 days of chronological aging (Fig. 1). Interestingly, although respiratory activity is slightly higher in CR cells after 6 h in culture, maximal respiratory rates are observed much later. Furthermore, the maximum rate of oxygen consumption in *S. cerevisiae* under CR is significantly higher than that observed in control cells, and occurs 12 h earlier (at 33 h for 0.5% and 45 h for 2.0%). Indeed, from the 24^th^ to the 42^nd^ h of culture, oxygen consumption by CR cells is increased when compared to control cells. This time period coincides with the use of ethanol and glycerol as carbon sources by CR cells (Table 1).

### 2. Substrate availability and consumption during chronological history are affected by CR and respiratory integrity

**Figure 2:**
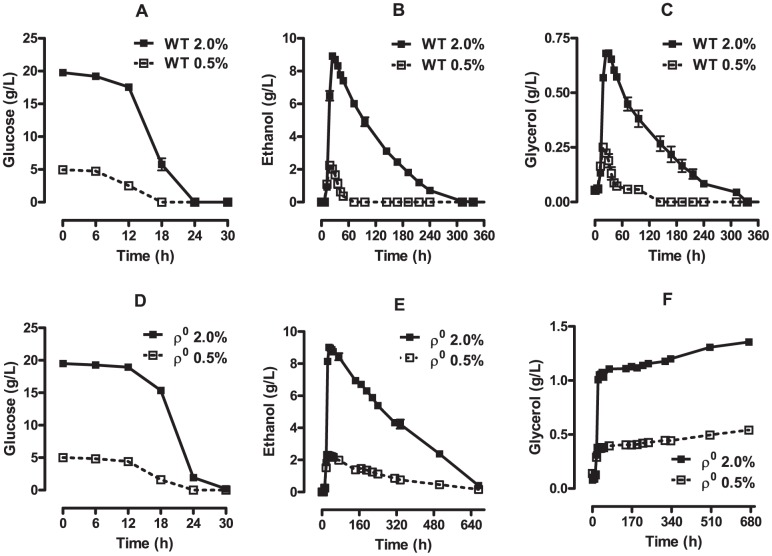
Substrate consumption during chronological aging in *S. cerevisiae*. Glucose (Panels A and D), ethanol (Panels B and E) and glycerol (Panel C and F) concentrations in the culture media during WT (Panels A-C) and ρ^0^ (Panels D-F) *S. cerevisiae* chronological lifespan were measured as described in Materials and Methods.

Differences in respiratory rates between control and CR cells have been attributed to the repressive effect of glucose on genes that encode proteins involved in aerobic metabolism, a phenomenon known as glucose repression [8], [13], [36]–[38]. Interestingly, however, glucose is exhausted from the culture media by the 24^th^ h for control conditions and 18^th^ h for CR (Fig. 2A, [39]). Thus, the most prominent changes in oxygen consumption rates observed in Fig. 1 occur many hours after no glucose can be detected in the culture media, indicating that cells grown under CR conditions hysteretically increase respiratory metabolism faster and to higher levels after glucose exhaustion. We questioned if this difference was due to a lower availability of oxidizable substrates for cells cultured under control conditions. However, measurements of ethanol and glycerol (Fig. 2B and C) indicate that levels of these substrates are higher in control cultures at these time points, and that they are exhausted much later than in CR cultures.

In order to determine the contribution of respiratory metabolism to the changes in substrate levels observed during chronological aging, we used respiratory-incompetent ρ^0^ cells (see Materials and Methods) and measured the levels of glucose (Fig. 2D), ethanol (Fig. 2E) and glycerol (Fig. 2F). As expected, ρ^0^ cells were capable of producing but not consuming glycerol (Fig. 2F), which is metabolized exclusively by oxidation. On the other hand, glucose consumption by ρ^0^ cells (Fig. 2D) was only slightly delayed relative to WT cells (Fig. 2A). Ethanol is also exclusively metabolized by oxidation, and, as expected, its decline was markedly decreased in ρ^0^ cultures (Fig. 2E) relative to WT (Fig. 2B). The slow progressive decline of ethanol in ρ^0^ cultures can be explained by its evaporation during 28 days of chronological aging.

### 3. Chronological aging is accompanied by drastic changes in media pH

Since oxidative metabolism varies strongly during chronological aging and with CR (Figs. 1–2), and *S. cerevisiae* growth conditions typically involve poorly buffered media, changes in pH are expected during chronological aging, and have been documented in the past [39], [40]. Indeed, we found that extracellular pH strikingly changed over time and in a manner strongly affected by CR (Fig. 3A). WT *S. cerevisiae* cultured under control conditions presented a pH of ∼5 throughout most of the 28 days in culture, while CR cells presented a strong increase in pH, especially during hours 144–240 (6–10 days). In addition, the pattern of these curves is influenced by the absence of mitochondrial DNA (Fig. 3B). Thus, respiratory metabolism is responsible for the large acidification of the medium in control cultures and alkalinization under CR conditions. Despite a strong contribution of respiratory metabolism toward changes in pH, a difference still is observed in ρ^0^ cultures under control and CR conditions. This is of interest, since CR does not extend chronological lifespan in ρ^0^ cells (Table 2, [13], [35]), suggesting that pH changes, *per se*, are not sufficient to induce enhanced *S. cerevisiae* lifespan. In order to test this hypothesis experimentally, we conducted experiments following chronological lifespan in cells in which the pH of cultures under control conditions was clamped at 7.5. As seen in Fig. 3C, maintaining the extracellular pH clamped, avoiding acidification, did not alter chronological survival in control cells. Thus, our results indicate that pH is not solely responsible for the increase in viability over time found in cells cultured under CR conditions.

**Table 2:** Chronological lifespan of WT and ρ^0^ cells cultured under control and CR conditions.

	WT		ρ^0^	
Day	Control	CR	Control	CR
**1**	78.71±4.85	102.7±5.62*	87.74±9.29	96.88±9.49
**7**	49.42±2.06	74.56±1.92*	56.41±8.80	75.77±14.00
**14**	42.36±2.55	73.00±3.29*	26.44±4.80	21.59±10.83
**21**	29.11±3.65	67.89±7.64*	14.75±2.37	7.442±1.29
**28**	22.99±3.18	60.02±2.37*	4.784±2.20	5.433±1.40

Values are expressed (means±SEM) indicate the number of colonies formed from 100 cells plated onto solid YPD media [13]. *p<0.05 versus control.

**Figure 4:**
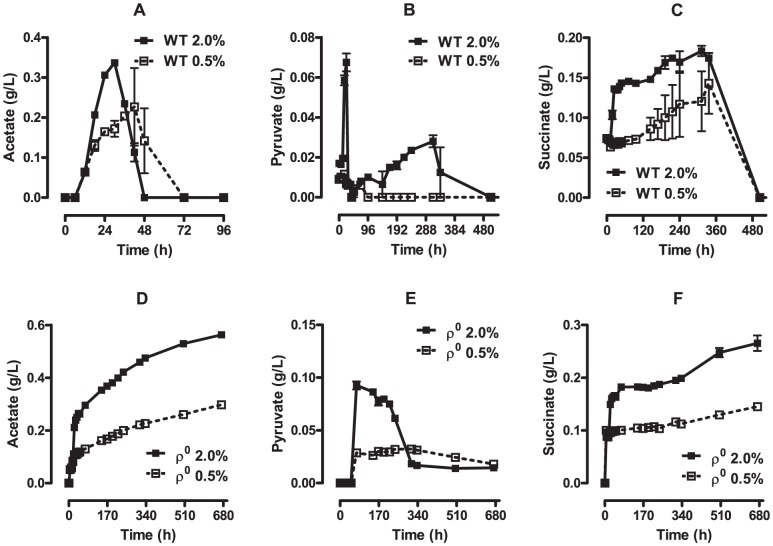
Acetate, pyruvate and succinate levels during chronological aging. Acetate (Panels A and D), pyruvate (Panels B and E) and succinate (Panel C and F) concentrations in the culture media during WT (Panels A-C) and ρ^0^ (Panels D-F) *S. cerevisiae* chronological lifespan were measured as described in Materials and Methods.

The avoidance of acetate formation and resulting acidification and toxicity has also been previously related to the increase of longevity associated with CR cultures ([39], but see ref. [9]). Accordingly, we measured acetate levels during chronological aging (Fig. 4A). We found evidence that acetate toxicity itself cannot directly account for the differences in pH between CR and control cultures, since it is only detectable in respiratory-competent WT cells before the 72^nd^ h (day 3) in culture, while pH differences persist throughout the 28 days. Indeed, large differences in the levels of other organic acids (pyruvate, Fig. 4B and succinate, Fig. 4C) could be observed between control and CR cells as they aged in culture, and differences in pH certainly reflect the added effect of many different metabolites. Furthermore, even when acetate consumption was absent in respiratory-incompetent ρ^0^ cells (Fig. 4D), the levels of this acid in the culture media did not surpass ∼0.6 g/L (10 mM), at least 10 times less than those found to affect cell survival [39].

### 4. CR and respiratory capacity affect biomass formation and the efficiency of energy conversion

**Figure 5:**
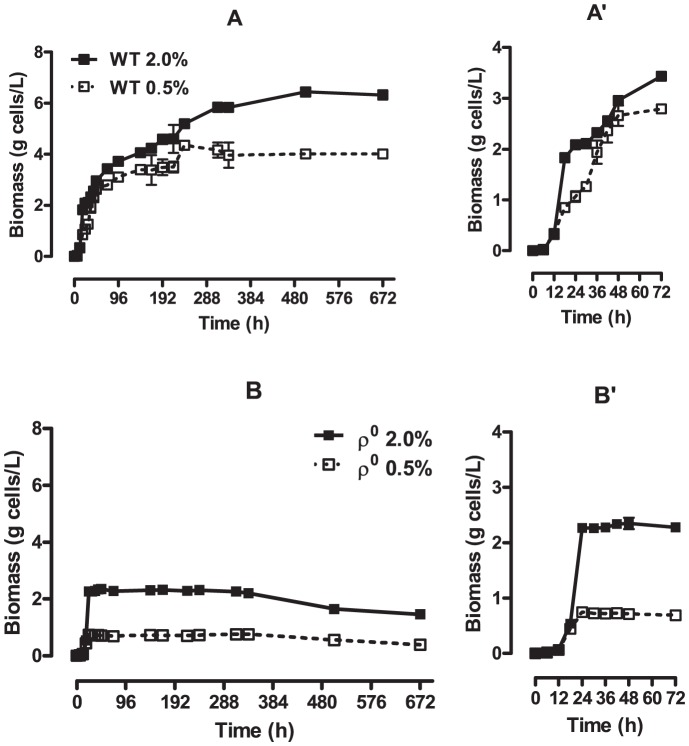
Biomass formation is altered by glucose levels and respiratory integrity. Biomass values during WT and ρ^0^ *S. cerevisiae* (Panels A-A’ and B-B’, respectively) chronological aging were measured as described in *Materials and Methods*. Panels A’ and B’ show a reduced time scale of the exponential growth phase.

We followed cell growth by measuring Abs_600_ of the culture media and determining the conversion factor between Abs_600_ and biomass, as described in *Materials and Methods*. We found (Fig. 5A) that the biomass formed after 28 days by WT cells cultured under CR conditions was 38.50±1.64% lower than that formed by control cells. This observation, together with the fact that the CR medium provides 75% less glucose, indicates that the efficiency of energy conversion exhibited by CR cells is increased.

Analyzing the biomass curves at a higher resolution (Fig. 5A’ and 5B’), a clear demonstration of the effect of mitochondrial DNA on the generation of biomass in the post-diauxic growth-phase [41] is seen: while WT cells exhibit growth promoted by the use of oxidizable substrates (Fig. 5A), ρ^0^ mutants do not (Fig. 5B), and no cellular growth is observed after the exhaustion of glucose (at ∼24 h, see Fig. 2D). Another difference is the maximum biomass formed by ρ^0^ mutants when compared to the WT strain (compare Figs. 5A and 5B): 55.42±0.92% lower. In addition, a 83.29±1.28% decrease in biomass formation by the ρ^0^ mutant is observed under CR conditions. This observation is consistent with the reduced availability of glucose in CR culture media and the inability to use aerobic substrates to generate biomass exhibited by ρ^0^ cells. The reduction of biomass in ρ^0^ cells observed from the 336^th^ h on (14^th^ day; Fig. 5B), both under control and CR conditions, may reflect the progressive increase in mortality and degradation of yeast cells [13]. Moreover, the increase in biomass presented by the WT strain, also from the 336^th^ h on (14^th^ day; Fig. 5A), can be explained by the evaporation of water from the system, an observable occurrence in prolonged culture conditions. Through measurements of 28 day incubated erlenmeyers, we found that water evaporation is ∼0.262 g per day.

### 5. Specific growth and glucose consumption are affected by respiratory capacity, but not CR

**Figure 6:**
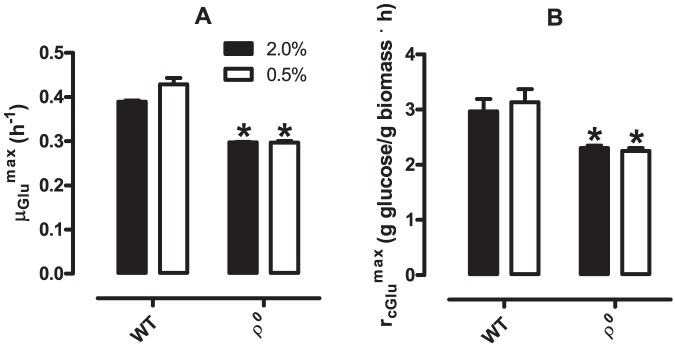
Specific growth rate and specific glucose consumption are decreased in ρ^0^ *S. cerevisiae* but unaltered by glucose levels. Specific growth rates in glucose (μ_glu_^max^, Panel A) and specific glucose consumption (r_glu_^max^, Panel B) of WT and ρ^0^ *S. cerevisiae* (as indicated) were calculated as described in *Materials and Methods*. *p <0.05 *vs.* WT (unpaired Student's *t* test).

Using the data presented in Fig. 2 and 5 and time intervals in which glucose was present in the media (Table 1), we were able to calculate specific cell growth rates on glucose (μ_Glu_^max^, Fig. 6A) as well as maximal specific glucose consumption rates (r_cGlu_^max^, Fig. 6B) for ρ^0^ and WT *S. cerevisiae* cultured under control and CR conditions. We found that CR does not alter specific growth on glucose (Fig. 6A), but that ρ^0^ mutants present a significant reduction, both under control and CR conditions. Similarly, maximal specific glucose consumption rates did not differ between control and CR WT cells (Fig. 6B) but the absence of mitochondrial DNA decreases r_cGlu_^max^.

### 6. Glucose-to-biomass, glucose-to-ethanol and glucose-to-glycerol conversion factors are not influenced by CR

**Figure 7:**
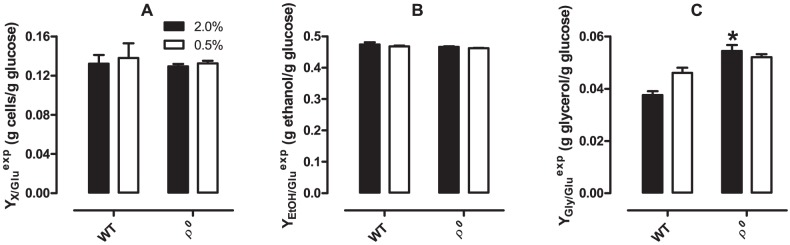
Glucose-to-biomass, glucose-to-ethanol and glucose-to-glycerol conversion factors. Glucose-to-biomass (Y_X/Glu_^exp^, Panel A), glucose-to-ethanol (Y_EtOH/Glu_^exp^, Panel B) and glucose-to-glycerol (Y_Gly/Glu_^exp^, Panel C) conversion factors in WT and ρ^0^ *S. cerevisiae* were calculated as described in *Materials and Methods*. *p <0.05 *vs.* WT (unpaired Student's *t* test).

We also determined glucose-to-cells (Y_X/Glu_^exp^, Fig. 7A), glucose-to-ethanol (Y_EtOH/Glu_^exp^, Fig. 7B) and glucose-to-glycerol (Y_Gly/Glu_^exp^, Fig. 7C) conversion factors during the time periods glucose was available in the culture media (Table 1). Interestingly, glycerol formation from glucose (Fig. 7C) was higher in ρ^0^ mutants compared to WT cells, and WT CR cells presented a trend (*p*  =  0.07) toward higher glucose-to-glycerol conversion compared to control cells. However, we found that ρ^0^ mutants do not exhibit reduced glucose to cell conversion (Fig. 7A) or increased glucose-to-ethanol conversion (Fig. 7B) when compared to WT cells. Additionally, CR does not alter these physiological parameters. This leads to the surprising conclusion that the ability to generate biomass and to form ethanol from glucose is independent of the integrity of the mitochondrial genome and is not changed by the amount of glucose available in the culture media at the beginning of the culture period.

### 7. Consumption, growth rates and conversion to biomass in respiratory substrates is increased in CR cells

**Figure 8:**
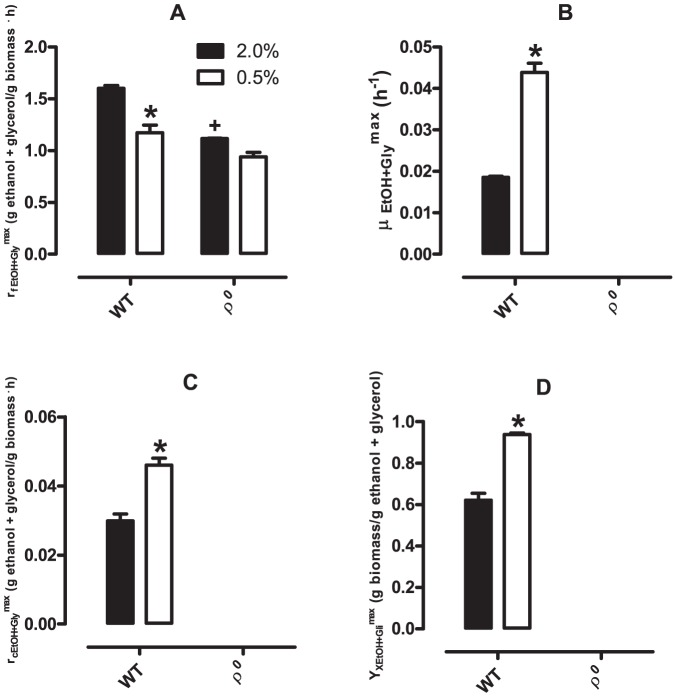
Maximum specific generation of ethanol/glycerol, specific growth rate in ethanol/glycerol, maximum specific consumption of ethanol/glycerol and ethanol/glycerol-to-cell conversion factors. Maximum specific generation of ethanol/glycerol (r_fEtOH+Gly_^max^, Panel A), specific growth rate in ethanol/glycerol (μ_EtOH+Gly_^max^, Panel B), maximum specific consumption of ethanol/glycerol (r_cEtOH+Gly_^max^, Panel C) and ethanol/glycerol-to-cells conversion factors (Y_XEtOH+ Gly_^max^, Panel D) were calculated as described in *Materials and Methods*. Panel A: *p <0.05 *vs.* 2.0%; +*p* < 0.05 *vs.* WT (unpaired Student's *t* test).

The lack of a change in glucose-to-cell conversion promoted by CR (Fig. 7A) associated with the higher efficiency of energy conversion observed in CR cells when measuring growth curves (Fig. 5) suggests CR increases metabolic efficiency with other substrates. Thus, we determined the conversion factors for respiratory substrates (Fig. 8). Since ethanol and glycerol consumption are temporally parallel (Fig. 4B and C), the maximum specific growth rate in ethanol and glycerol could not be separately obtained, and the calculation of the formation, consumption and conversion factor to cells of both substrates was performed together, at the time intervals depicted in Table 1.

Fig. 8 shows maximum specific ethanol/glycerol formation rate (Panel A), maximum specific cell growth rate on ethanol/glycerol (Panel B), consumption of ethanol/glycerol (Panel C) and the conversion of these substrates into cells (Panel D). We found that ethanol/glycerol formation is reduced both by CR and the functional absence of mitochondrial DNA (Fig. 8A). In ρ^0^ cells, which are not capable of using ethanol and glycerol as carbon sources, no increase in biomass was observed supported by these substrates (Fig. 8D). Furthermore, we found that the specific growth rate on ethanol/glycerol is higher in CR cells than controls (Fig. 8B). This observation is consistent with the significant increase in both ethanol/glycerol consumption (Fig. 8C) and conversion to biomass (Fig. 8D) in CR *S. cerevisiae*. Since we found that glucose-to-biomass conversion is equal between WT control and CR cells (Fig. 7A), the larger efficiency of ethanol/glycerol-to-biomass-conversion (Fig. 8D) in CR cells explains the higher overall energy conversion observed under CR discussed under point 4.

**Figure 9:**
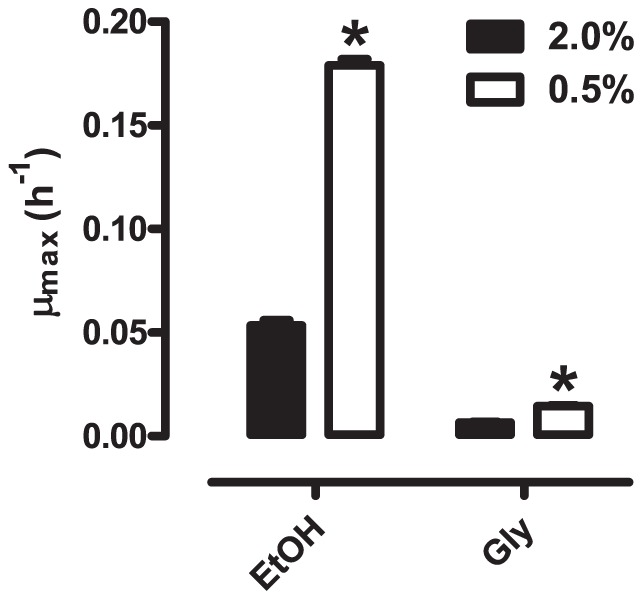
Specific growth rates in ethanol or glycerol. Specific growth rates in ethanol (EtOH) or glycerol (Gly) were determined as described in *Materials and Method*, with cells removed from their original media and added to media containing each of these substrates (8.98 g/L ethanol and 0.68 g/L glycerol). *p < 0.05 vs. 2.0% (unpaired Student t-test).

Since it was not possible to separate the contributions of ethanol and glycerol in the growth of WT cells under control and CR conditions, we cultured cells in standard YPD until the maximal concentrations of ethanol and glycerol were achieved (see Fig. 2B and C). The cells were then transferred to media in which ethanol or glycerol were the only substrate, present at the maximal concentration they reach in culture (8.98 g/L for ethanol and 0.68 g/L for glycerol), and the specific growth rates for ethanol and glycerol were determined (Fig. 9). Growth rates under these conditions were higher than those in native growth media (compare Figs. 8B and 9), possibly due to the refreshed yeast extract and peptone. Despite this, we were able to verify that, under control and CR conditions, growth in ethanol is much higher than in glycerol. In addition, we verified that CR cells presented substantially larger growth rates when compared to control cells with both respiratory substrates. This confirms that CR culture conditions hysteretically prime cells toward better growth in respiratory media, long after glucose is exhausted.

In order to verify if the effect of CR on respiratory metabolism of ethanol is central toward its lifespan, we compared the survival of CR and control cells with and without respiratory activity (Table 2). As observed previously [13], [35], respiratory-incompetent ρ^0^ cells do not present increased survival under CR conditions, despite the fact that their lifespan is much longer than 24 h, a time point after which glucose cannot be detected in the media. This result confirms that enhanced respiratory metabolism promoted by CR is necessary for extended lifespan promoted by glucose limitation.

## Discussion

We evaluated here how oxidative energetic metabolism is affected by CR during chronological aging, based on the fact that increases in respiratory rates have been pinpointed as essential for the lifespan-expanding effects of CR [13]–[17], [35]. Interestingly, we found that, while cellular respiration is slightly higher in CR cells during the early hours in culture - as had been previously observed [10], [32] - the effect of CR on respiration is much more marked between 24 and 74 h (Fig. 1). This finding is remarkable, since it demonstrates that the most notable effects of CR on oxidative metabolism occur long after glucose is totally exhausted from the media, characterizing a hysteretic effect.

Hysteresis, or the dependence of a biological response not only on the current environment but also on the past environment, is involved in the control of many cellular functions including the modulation of the cell cycle [42]. Therefore it seems consistent to find that it is also implicated in the control of respiration during chronological lifespan. Indeed, we found that CR hysteretically primes cells to respond earlier and more intensely with an increase in respiratory rates during aging (Fig. 1). Although we do not yet know what signaling mechanisms mediate this hysteretic effect of CR, it is central toward the lifespan expanding effects, since multiple different interventions generating respiratory-incompetent cells result in a lack of response to CR (Table 2, [13], [35]).

A result of enhanced respiratory metabolism in *S. cerevisiae* is the change in production of acidic products associated with alterations in media pH. This is especially relevant in yeast cultures, which are generally poorly buffered. Indeed, it has been proposed that the shift in pH and, specifically, prevention of acetate formation, is the mechanism through which CR increases chronological lifespan [39], [40]. Although we see large changes in pH over time and under CR or control culture conditions (Fig. 3), our results suggest this is not the sole direct mechanism responsible for limited cell survival under control culture conditions, for a series of reasons: (i) CR is ineffective as a lifespan extension intervention in ρ^0^ cells (Table 2, [13], [35]), yet pH in the culture media is significantly different under these conditions (Fig. 3B); (ii) buffering pH does not eliminate lifespan extension promoted by CR (Fig. 3C) and (iii) differences in pH between CR and control WT cultures peak around 200 h (Fig. 3A), however, most significant differences in lifespan occur much later (Table 2). Conversely to our data, other groups have found that buffering media pH to 6.0 in control cells increases chronological lifespan [40], [42], [43]. One important difference in these studies is the use of synthetic complete medium, which is more poorly buffered (cultures reach pH as low as 2.5 [39]) and supports more limited survival than YPD. Furthermore, the effects of buffering pH in 2% glucose cultures were not compared to those of CR in these studies, and it is therefore not possible to conclude if pH buffering was sufficient to promote the full effects of CR under those conditions. While we certainly believe that acidic pH is toxic to cells, our results indicate that buffering extracellular pH is not sufficient to induce the fully extended lifespans observed in CR, a concept in line with previous data showing that acidification of CR cells is not sufficient to decrease lifespan [18]. For a two-sided review on the ongoing debate regarding the effects of pH in yeast lifespan and CR, we recommend reference [9].

Our results also suggest that direct acetate toxicity is not responsible for the differences in survival under CR and control conditions [9], [39] since: (i) it was undetectable in WT cells under both culture conditions after 72 h; (ii) the levels of this acid in the culture media did not surpass ∼0.6 g/L (10 mM), while levels of 200 mM are necessary to promote a decrease in *S. cerevisiae* viability [39]; (iii) acetate levels differ significantly between CR and control ρ^0^ cell cultures (Fig. 4D), but CR does not extend lifespan in these cells (Table 2, [13], [35]). It should again be noted that previous experiments that suggest the central role of acetate in CR lifespan extension [39] were conducted in synthetic media, while our results are in YPD, which is less prone to dramatic pH changes. Again, we recommend reference [9] for further insight into the ongoing debate on the role of acetate in chronological lifespan extension by CR.

Another often-proposed lifespan-extending effect discussed for the yeast CR model has been the shift toward oxidative metabolism of glucose [10], [17]. Very surprisingly, our results show this is not the case. Maximum specific growth rates on glucose (Fig. 6A), glucose specific consumption rates (Fig. 6B), glucose-to-cell conversion (Fig. 7A) and ethanol production from glucose (Fig. 7B) were unaffected by CR. Taken together, these data demonstrate that the ability of cells to metabolize and grow on glucose, as well as the proportion of ethanol produced per glucose molecule metabolized, is identical under both growth conditions. Thus, our results clearly indicate, through different but highly consistent findings, that glucose is predominantly fermented under both CR and control conditions, and that CR does not stimulate the respiratory metabolism of glucose in the early hours in culture.

A higher biomass production observed under control conditions versus CR (Fig. 5A) was observed, but it is explained by the total glucose available in the culture media, not a higher efficiency in use of energy (which is in fact lower, Fig. 8C). Likewise, the increased total amount of ethanol generated by cells in control media (Fig. 2A) is also explained by the higher initial concentration of glucose. Again, these results show that CR does not alter glucose metabolism, which is predominantly fermentative (as indicated by similar quantitative results obtained with ρ^0^ cultures).

Although glucose-to-cell conversion (Fig. 7A) and glucose-to-ethanol production (Fig. 7B) were unaffected by CR, WT cell growth in ethanol/glycerol (Fig. 8B), ethanol/glycerol consumption (Fig. 8C) and conversion to biomass (Fig. 8D) are all significantly enhanced under CR conditions. This indicates that, while CR does not enhance the respiratory metabolism of glucose, it increases the speed and efficiency of use of exclusively respiratory substrates. The effect is seen for both ethanol and glycerol, although experiments using the substrates separately (Fig. 9) indicate that the change in specific growth in ethanol is far more substantial. These results again demonstrate a hysteretic effect of CR, which primes cells to utilize respiratory substrates faster and more efficiently, after the complete elimination of glucose from the culture media.

Interestingly, this hysteretic effect is intrinsic of the cells, since it persists in substituted media (Fig. 9). Furthermore, since CR is ineffective in cells that are not capable of respiring (Table 2, [10], [13], [14], [35]), we propose that the hysteretic preparedness for earlier, faster and more efficient oxidative metabolism of ethanol is central toward the lifespan-enhancing effects of CR.

## Conclusions

We demonstrate here that decreased glucose availability (CR) in *S. cerevisiae* promotes:

1. Earlier and significantly more prominent increases in oxygen consumption relative to control cells;
2. A sustained difference of more than 3 extracellular pH units in the culture media, due in part to respiratory metabolism, and which cannot be attributed to the generation of a single acidic metabolite;
3. Increased efficiency of growth in respiratory substrates and metabolism of ethanol and glycerol, without changes in the metabolism of glucose.

Overall, our results suggest that CR hysteretically primes aging *S. cerevisiae* toward earlier, faster and more efficient metabolism of exclusively respiratory substrates, and that this effect is central toward lifespan enhancement promoted by CR.
